# Electrocardiographic Changes in Jordanian Patients With Becker Muscular Dystrophy

**DOI:** 10.7759/cureus.47553

**Published:** 2023-10-24

**Authors:** Mohammed K Al-Raqad, Shorouk Alwahsh, Issa S Hejazi, Osama T Abu-Salah, Lina Alshadfan, Amal Abu-Ledeh, Nour Ghanem, Lana Braik, Ahmad D Raggad

**Affiliations:** 1 Genetics, Al-Balqa Applied University, Al-Salt, JOR; 2 Pediatrics, Al-Balqa Applied University, Al-Salt, JOR; 3 Cardiology, Royal Medical Services, Amman, JOR; 4 Neurology, University of Jordan, Amman, JOR

**Keywords:** q wave, dilated cardiomyopathy, cardiac involvement, electrocardiograph, becker muscular dystrophy

## Abstract

Background and aim

Becker muscular dystrophy (BMD) is an X-linked disease caused by an in-frame mutation in the dystrophin gene, which is considered an allelic disorder to the most severe form of dystrophinopahies, Duchenne muscular dystrophy, which leads to skeletal and cardiac muscle involvement and results in dilated cardiomyopathy (DCM). The aim of this study is to present our ECG data and the significance of this data in the early detection of DCM in these patients.

Methods

This is a retrospective study. All patients known to the clinical Genetic Clinic and Queen Alia Heart Center in Jordan with a diagnosis of Becker muscular dystrophy from the year 2011-2022 are offered cardiac evaluation according to the guidelines, which included clinical assessment, electrocardiograph, and 2-D echocardiograph (echo)* *at the time of diagnosis and every five years thereafter once the initial assessment was normal. All the records were retrieved and analyzed.

Results

Fifty-three patients of all ages with genetically confirmed BMD were identified. Twelve had no record as they didn't attend any cardiac evaluation. Forty-one were under regular clinical follow-up. Two were excluded as they died, and another four had no recorded data in our center. Ultimately, 35 patients were included and studied. The mean age was 30.5 years ± 22.1, ranging from two to seventy-seven years of age. Twenty-seven (77%) had abnormal ECG. High voltage R wave in V2 and V1 was the most common finding, followed by repolarisation abnormalities and Q wave (43%, 17%, 13%, and 11% respectively). Incomplete right bundle branch block in 4% as well as R/S ratio >1.2. U wave abnormalities in 3% and sinus tachycardia were found in only one patient.

Conclusion

Cardiac surveillance for patients with Becker muscular dystrophy is mandatory after the age of 16. Q wave and repolarisation changes should be taken seriously as early signs of dilated cardiomyopathy, even if the echo is normal.

## Introduction

Becker muscular dystrophy (BMD), according to the Online Mendelian Inheritance in Man (OMIM #300376), is an X-linked recessively inherited disorder caused by a mutation in the dystrophin gene. BMD is considered as an allelic disorder to Duchenne muscular dystrophy (DMD) and X-linked dilated cardiomyopathy (XLDC). These disorders are characterized by alteration of the dystrophin gene product, dystrophin protein, a structural protein important for maintaining the integrity of cardiac and skeletal cytoskeleton. In cases of BMD, the alterations are usually due to in-frame deletions, duplications, or point mutations in the dystrophin gene. BMD was first recognized as distinct from DMD by Becker in 1955. The age of onset varies widely, ranging from two to 35 years, and there are reports of cases with even later onset. BMD is characterized by progressive proximal muscle weakness, starting in the lower limbs and progressing to involve the trunk and upper extremities with high plasma creatine phosphokinase (CPK) levels. Patients typically develop a waddling gait between the ages of 10 and 15 years, pseudo-hypertrophy of affected muscles by age 20, and inability to undertake manual work or climb stairs by age 30 [1]. Facial muscles remain unaffected. Other clinical features can include cognitive impairment related to loss or dysfunction of an isoform of dystrophin (Dp140) expression in the brain, found in 25% of BMD patients [2], and respiratory dysfunction [3].

A correlation between genotype and phenotype in dystrophinopathy has been studied, and one group demonstrated that "C-terminal domain mutations were associated with decreased wheelchair use and increased forced vital capacity. Dp116 and Dp71 mutations were also linked with decreased wheelchair use, while Dp140 mutations significantly predicted cardiomyopathy" [4].

Dilated cardiomyopathy (DCM), as seen in Duchenne muscular dystrophy (DMD), has been a well-recognized complication for a long time. Crucially, cardiac involvement (CI) may dominate the clinical picture and be out of proportion to skeletal muscle involvement [5-7] and can even occur in the absence of muscle symptoms [5,8]. The onset of left ventricular systolic dysfunction can be detected by several methods, including echocardiography, radionucleotide ventriculography, cardiac CT, or MR imaging. The addition of tissue Doppler measures to conventional echo measurements increased the sensitivity of the test, and dobutamine-stress echocardiography is a further sensitive method for detecting reduced cardiac systolic reserve. The principle of stress echocardiography is to examine changes in regional wall motion behavior in response to increased heart rate and inotropic stimulation. Although still not widely available in Jordan, cardiac MR imaging (cMRI) is a highly sensitive, non-radiation-dependent way of assessing cardiac structure and function non-invasively [9-11]. By using gadolinium-enhanced cMRI, it is also possible to identify, localize, and characterize areas of myocardial fibrosis. Up to 75% of BMD patients have abnormal cardiac findings on more sensitive assessments [12]. Heart failure, sometimes complicated by arrhythmias, is thought to be a terminal event in about 50% of BMD patients [13]. In addition to direct left ventricular dysfunction, there is evidence of autonomic nervous system imbalance in patients with BMD. The pattern is of sympathetic predominance with faster resting rates and lower heart rate variability [14]. Some have reported wider QT-interval dispersion in BMD and suggested that this might contribute to their risk of sudden death [15]. On surface 12-lead Electrocardiograph (ECG), the most frequently observed abnormalities are abnormally tall R-waves in leads V1-2 and pathological Q waves in inferoapical (II, III, AVF, V5-6) or lateral (I, aVL) leads. These abnormalities are thought to reflect epicardial fibrosis in the respective segments of the left ventricle. Although much has been written about cardiac involvement in Duchenne muscular dystrophy (DMD), there is a paucity of similar reports in patients with BMD.

We believe this is the first report from Jordan studying cardiac involvement in Becker muscular dystrophy, so the aim of this study is to present our ECG data and the significance of this data in the early detection of DCM in these patients.

## Materials and methods

This study was conducted in a retrospective manner with a waiver of written informed consent.

Inclusion and exclusion criteria

All male patients with a diagnosis of BMD and under follow-up at the clinical genetic unit at Al-Balqa Applied University's Hospital (Al-Husein Hospital, AL-Salt, Jordan) and Queene Alia Heart Center, Royal Medical Service, and Amman-Jordan were included. Duchenne muscular dystrophy (DMD) and other neuromuscular disorders were excluded.

All BMD was ascertained at the clinical genetic units by medical history, physical examination, creatine kinase (CK) level, muscle biopsy with immune-histochemistry (IHC), and molecular investigations demonstrating reduced dystrophin staining by IHC on muscle biopsy. DNA was analyzed by multiplex ligation-dependent probe amplification (MLPA; MRC Holland, Amsterdam, Netherlands, kits P034-B2 and P035-B1), which was the first line method to identify exonic deletion/duplication in the 79 exons of the dystrophin gene. In case MLPA returned negative, this was followed by direct sequencing of the dystrophin gene or whole exome sequencing utilizing next-generation sequencing (NGS) to detect a point mutation.

All BMD patients were offered annual cardiac assessments as part of their multidisciplinary care according to our institute guidelines. Each assessment consisted of an inquiry regarding muscle and cardiac symptoms, a 12-lead ECG, and a trans-thoracic echocardiogram.

The medical records of all patients in the study were reviewed by a cardiologist, a neurologist, a pulmonologist, and a clinical geneticist.

12-lead electrocardiograms (ECG)

ECGs were analyzed for the presence of pathological Q wave, defined as greater than one-third of succeeding R wave, >0.04 mm wide or >0.2 mV deep in inferior or lateral leads, abnormal repolarization changes, such as dysmorphic or inverted T-waves, or abnormally tall R-wave voltages in precordial leads V1-2. Abnormal T-waves were defined by discordance between QRS and T-wave orientation and/or the presence of U-waves in adults. R-wave greater than 7 mm in adults and >19 mm in children below the age of 16 years or an R/S ratio >1.2 in leads V1-2 [10]. ECGs were also examined for the presence of AV or bundle branch blocks (BBB) and ectopy or more sustained arrhythmias. The cardiomyopathic index - defined as QT / PQs interval ratio - was not measured in this study, because its clinical significance is controversial. This index has been proposed as evidence of the pre-clinical or 'electrical' stage of cardiomyopathy [16,17].

## Results

**Figure 1:**
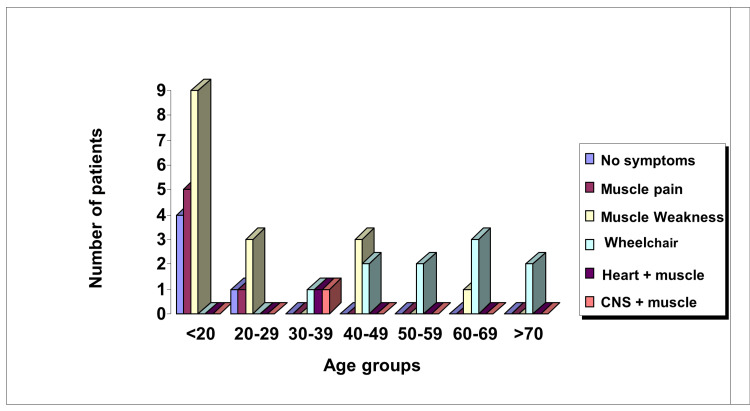
Clinical picture in Becker muscular dystrophy patients (n=39)

Fifty-three patients of all ages with genetically confirmed BMD were identified. The mean age was 30.5 years ± 22.1, ranging from two to seventy-seven years of age. Twelve had no record as they didn't attend any cardiac evaluation. Two were excluded as they died. Thirty-nine were under regular cardiac surveillance. Five out of the original thirty-nine patients (13%) were asymptomatic, while 34 out of 39 (87%) were suffering from skeletal muscle symptoms with varying degrees from muscle pain to wheelchair-bound. One patient had heart symptoms in the form of short-lived palpitation, and another one had central nervous system (CNS) manifestations (Figure 1).

Four out of the 39 had no recorded data in our Heart Center as they were being followed in the private sector or were under the age of five years. Ultimately, 35 patients were included and studied.

Follow-up with ECG and echo was available for 27 with a mean interval between assessments of 5.4 + 4.3 ranging from 0.7 to 20.2 years. No one came with symptoms of heart failure. The mean age at initial assessments was 30.37 + 21.49, ranging from two to 77 years. Five patients had one or more co-morbid conditions (hypertension, diabetes mellitus (DM), bronchial asthma, depression, and complete atrioventricular (AV) block). Fourteen patients (40%) patients had abnormal echo findings. DCM was diagnosed in 13 of them (37%) based on the following criteria: the presence of dilated heart, wall motion abnormality, and impaired left ventricle (LV) function either by reduced fractional shortening (FS) and/or ejection fraction (EF) below 28% and 60%, respectively. The mean age of onset of DCM was 37 years (range 17-76 years).

ECG findings

Thirty-five out of thirty-nine (90%) had 12-lead ECG records. Among the four with no ECG, three due to young age and one who was followed in another hospital, and we didn't have access to the record. Overall, 27 (77%) of 35 patients had at least one abnormal ECG (Table 1).

**Table 1:** Summary ECG findings in 39 BMD patients R/V1-2 normal value - <28 mm (3-8 years), <19 mm (8-16 years), <7mm (adult); R/S normal value <1.2; Q wave: <0.04 second or < 1/3 of R wave. iRBBB - incomplete right bundle branch block, NA - not available, Lft - left, Rt - right, BMD - Becker muscular dystrophy

Case no.	Age (yrs)	HR	R-wave in V_1_	R/V2	R/S ratio	Q wave	T-wave abnormalities	iRBBB	U wave
1	2	NA	NA	NA	NA	NA	NA	NA	NA
2	4	NA	NA	NA	NA	NA	NA	NA	NA
3	5	NA	NA	NA	NA	NA	NA	NA	NA
4	5	75	No	No	Normal	No	No	No	No
5	9	59	No	No	Normal	No	No	No	No
6	12	76	No	No	> 1.2(V-2)	No	No	No	No
7	13	73	No	20 mm	Normal	No	No	No	No
8	13	64	No	No	Normal	Lateral leads	No	No	No
9	13	81	No	No	Normal	No	Septal(V1-V2)	No	No
10	13	71	No	No	Normal	Lateral leads	Lateral leads	No	No
11	14	65	No	21 mm	Normal	Right precordial	No	No	No
12	16	62	21 mm	24 mm	> 1.2 (V1)	Lateral leads	Diffuse	yes	No
13	17	75	No	No	Normal	No	No	No	No
14	17	89	13 mm	17 mm	Normal	Inferior leads	No	No	No
15	17	64	No	10 mm	Normal	No	No	No	No
16	17	76	No	11 mm	Normal	No	Diffuse	Yes	Right Precordial
17	18	78	No	No	Normal	No	No	No	No
18	18	72	12 mm	13 mm	Normal	No	No	No	No
19	22	85	No	14 mm	Normal	No	Inferior leads	No	No
20	22	46	No	12 mm	Normal	Lateral leads	Left. Precordial	No	No
21	24	73	No	No	Normal	No	No	No	No
22	27	66	8 mm	8 mm	Normal	No	Diffuse	No	Antero-septal
23	28	64	No	No	Normal	Lateral leads	Right precordial	No	No
24	34	80	Yes	No	Normal	Lateral leads	Inferior	No	No
25	35	76	No	No	Normal	No	No	No	No
26	39	69	No	No	Normal	Infero-lateral	Right precordial	No	Right precordial
27	42	71	No	9 mm	Normal	No	No	No	No
28	42	70	No	No	Normal	No	No	No	No
29	42	60	No	No	Normal	No	Infero-lateral	No	No
30	44	73	No	15 mm	Normal	No	No	No	No
31	47	74	No	8 mm	Normal	Inferior lead	Anterior	No	No
32	51	NA	NA	NA	NA	NA	NA	NA	NA
33	57	84	13 mm	13 mm	> 1.2(V1-2)	Lateral leads	No	Yes	No
34	62	105	No	No	Normal	No	No	No	No
35	63	70	No	No	Normal	No	No	Yes	No
36	66	76	No	No	Normal	No	No	No	No
37	67	75	No	No	Normal	No	No	No	No
38	76	68	No	No	Normal	No	Diffuse	No	No
39	77	76	No	9 mm	> 1.2(V-2)	No	No	No	No

**Figure 2:**
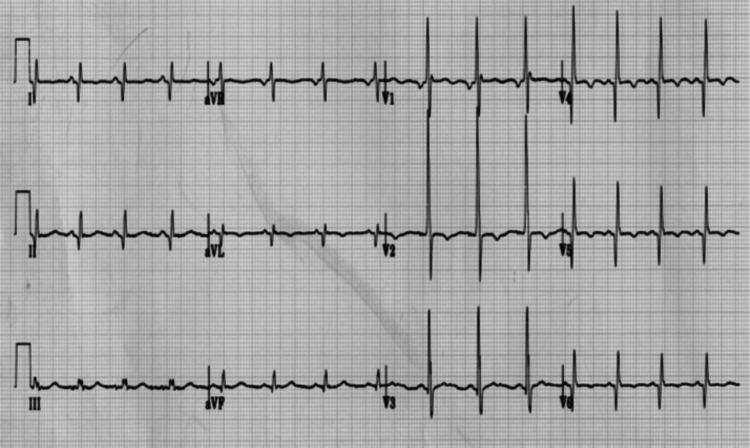
ECG changes in affected male with cardiac dystrophinopathy Short P-R. Q waves in leads I, II, and V4-6. Abnormally tall R in V1-2. T wave inversion in V2, V 4-6.

The mean resting heart rate was 72.6 + 9.96 beats per minute. Sinus tachycardia (>100 bpm), commonly seen in DMD patients, was observed in only one BMD subject. Abnormally large R-wave amplitude was seen in the right precordial leads (V1 and V2) in 21 (60%) subjects. Abnormal repolarization changes were seen in 16 (46%), including T-wave abnormalities in 13 and prominent U-waves in three patients. The R/S ratio was abnormal in 4 (11%). Conduction defects in the form of iRBBB were found in 4 (11%). Pathological Q wave was seen in 11 (31%): laterally in seven, inferiorly in two, infero-laterally in one, and in the right precordial leads in one (Table 2, Figure 2).

**Table 2:** Summary of ECG abnormalities in BMD patients (total number= 35) BMD - Becker muscular dystrophy, RBBB - right bundle branch block R/V1-2 normal value - <28 mm (3-8 years), <19 mm (8-16 years), <7mm (adult); R/S normal value <1.2; Q wave: <0.04 second or > 1/3 of R wave.

Abnormal ECG findings	Number (%)
R/V1	6 (17%)
R/V2	15 (43%)
R/S ratio	4 (11%)
Incomplete-RBBB	4 (11%)
Pathological Q waves	11 (31%)
T wave abnormalities	13 (37%)
U wave	3 (9%)
Sinus tachycardia (>100 bpm)	1 (3%)

## Discussion

Cardiac involvement (CI) in BMD can be diagnosed upon clinical examination, ECG, ambulatory ECG, echocardiography, cardiac MRI, and endomyocardial biopsy [18]. Treatment depends on the stage of the disease, either asymptomatic or in heart failure, and comprises the following: diuretics, digitalis, angiotensin-converting enzyme inhibitors, β-blockers, and anticoagulants. Pacemaker implantation and implantable cardioverter defibrillator can be used in some cases. Heart transplantation is considered in cases of intractable heart failure [19]. Stretch-induced damage contributes to muscle fatigue and weakness mediated by changes in intracellular calcium, sodium, and pH. This mechanism, which is involved in normal muscle fiber damage, is believed to be exaggerated in cases of muscular dystrophy [20]. In cases of dystrophinopathies, as in BMD, the altered dystrophin protein leads to the replacement of myocardium by connective tissue and fat, which is more prominent in the posterobasal and lateral walls of LV [13]. The limitation of compensatory cardiomyocyte hypertrophy, besides the progressive loss of cardiomyocyte in DMD/BMD patients, contributes to progressive clinical cardiac course in these patients.

CI could be subclinical without any signs or symptoms of heart problems or clinically evidenced by the presence of these symptoms. CI is considered when any of the characteristic ECG or echo findings are present in the examined subject. In our study, ECG abnormalities were found in 27 (77%) of the patients, which is close to other authors' findings (Table 3) [3, 21-23].

**Table 3:** Comparison of abnormal cardiac findings between our study and other authors DCM - dilated cardiomyopathy

Authors	N studied	ECG abnormalities	Echo abnormalities	DCM
Xiong et al. [3]	25	76%	-	-
Melacini et al. [22]	31	68%	62%	39%
Yilmaz et al. [23]	15	47%	53%	47%
Hoogerwaard et al. [21]	27	71%	55%	33%
Current study	35	77%	40%	37%

High voltage R wave in V2 and V1 was the most common, followed by repolarization abnormalities and Q wave. The Q wave is assumed to represent selective atrophy and scarring of the posterobasal region and adjacent lateral wall. In contrast to abnormal ECG findings in DMD, where sinus tachycardia is commonly seen, our results showed it occurred in only one patient. All the patients with DCM had abnormal ECG findings, whereas fifteen with abnormal ECG didn't show any evidence of DCM by echo. This means that ECG changes could be the first sign of evolving DCM, and these patients need close follow-up as they are more likely to develop DCM. IRBBB was found in four patients: two of them with a normal heart. Ten out of the 21 with high voltage R wave in V1 or V2 didn't show any signs of DCM, while three out of four with R/S ratio more than 1.2 proved to be normal. On the other hand, seven out of eleven with Q wave and four out of fourteen with repolarization changes proved to have DCM. The first ECG changes occurred early in the course of the disease (from 12 to 17 years) with an R/S ratio greater than the normal value seen as early as twelve years. Repolarization changes and the presence of Q wave seem to be more sensitive than other ECG parameters in predicting DCM, but the presence of any abnormal ECG findings should be considered as an early alarming sign for early evolving DCM.

**Figure 3:**
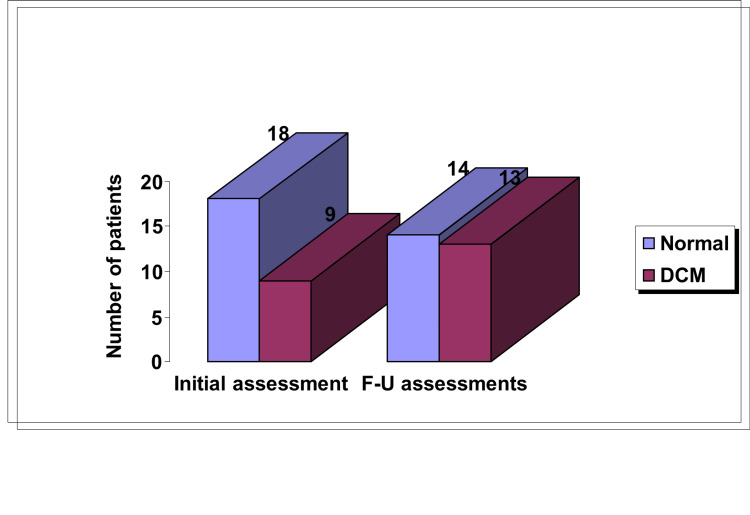
Progression of dilated cardiomyopathy in Becker muscular dystrophy patients during follow-up DCM - dilated cardiomyopathy

DCM was diagnosed in thirteen patients (37%) based on echo findings, which is similar to other authors' conclusions (Figure 3 and Table 3). Xiong et al. [3] studied 25 patients with BMD and reported that 76% had ECG abnormalities, but they didn't comment about DCM. While Melacini et al. [22] found that of 31 BMD patients, 39% had DCM. In addition, Hoogerwaard et al. [21] studied 27 BMD patients and found 33% of them had DCM, and 47% was reported by Yalmaz et al. [23] after studying 15 BMD patients. The minor differences between these reports could be related to the different parameters used and to the threshold for defining abnormality.

Wall motion abnormalities, which are considered a reflection of regional scarring and fibrosis, were consistent in all patients with DCM apart from one patient who was found to have impaired LV function besides dilated heart and characteristic ECG changes.

No one of the thirteen patients with DCM was asymptomatic, and the symptoms ranged from mild muscle aches to wheelchair dependent. There was no correlation between the severity of muscle and heart involvement, and this pattern of clinical picture was reported by other authors [19]. No one with DCM presented with cardiac symptoms, and the only patient with cardiac symptoms (palpitation) had a normal heart.

Follow-up data were available for 27 patients, which enabled us to comment on the course of the disease and the response to treatment. The progressive nature of CI in BMD was evidenced in our study, as four of the thirteen patients with DCM were reported to be normal in the initial assessment (Figure 3). This is in keeping with the natural history of cardiomyopathy in affected males with dystrophinopathy that followed a progressive nature from preclinical involvement to dilated end-stage heart failure, passing a stage of hypertrophy.

**Figure 4:**
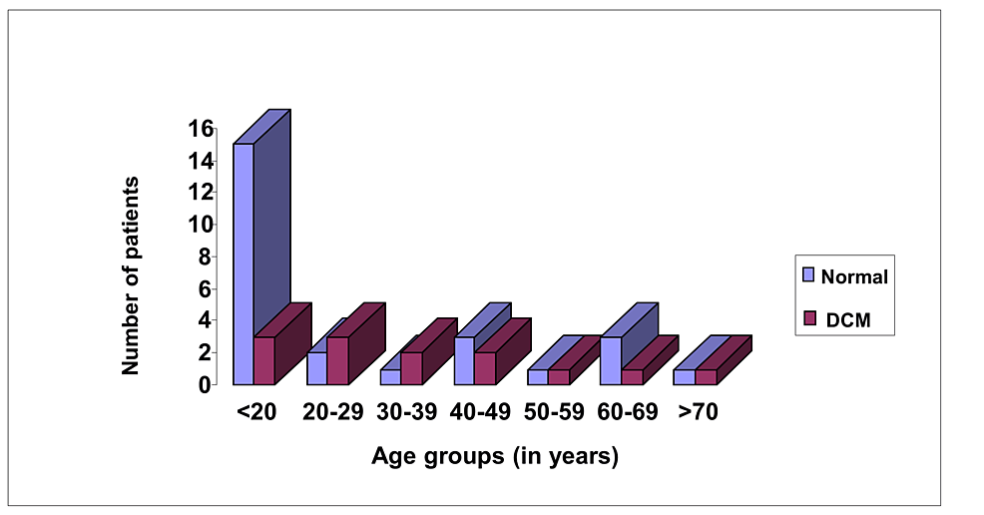
Age of onset of cardiomyopathy in 39 Becker muscular dystrophy patients DCM - dilated cardiomyopathy

The mean age of onset of DCM and the commencing treatment was 36.55 + 18.73 years, ranging from 17 to 76 years (Figure 4). This means the DCM can appear at any time, but no one developed DCM below the age of 16 years despite the ECG findings being seen early in the first decade of life. This finding was supported by other author's results that cardiac function is well preserved in these patients below the age of 13 years [24, 25]. On the other hand, one report showed that DCM can be manifested as early as 11 years of age [26].

Whether ECG and echocardiograph are considered enough tools in screening for CI in BMD or not should other advanced methods such as cardiac MRI be employed have yet to be answered. The echocardiographic LV function variables FS and EF are derived from linear measurements in one dimension and reflect global LV function in symmetrically contracting ventricles, and this is not the case in dystrophinopathy where regional scarring is the predominant presentation rather than global involvement and hence asymmetrical contraction for that it may seem inappropriate to use such parameters in BMD. In addition, the posterior wall is progressively involved as the disease progresses over time; this will result in a reduction of contractility. However, poor echo windows in some patients with dystrophinopathy due to muscle weakness and scoliosis can limit interpretations of echo findings despite the feasibility of such tests and good compliance by patients in different age groups. In addition, standard echo couldn't detect the reserved heart function, while stress echo is considered superior in this aspect as we can test the viability of the myocardium during exercise. The one patient from our cohort who underwent Dubtamine stress echo showed good cardiac reserved function, and his EF returned to normal, but his chronotropic response was under the expected level, which pointed to autonomic imbalance. Cardiac MRI so far is not routinely included in our services; thus, none of our cohort underwent cMRI. cMRI is believed to be a good tool to pick up cardiac abnormalities superior to echo, especially for regional analysis of the myocardium and providing more detailed anatomical and structural information. Aikawa et al. [10], which is supported by other authors' findings, showed that the detection rate of any abnormal cardiac finding could reach up to 80% by cMRI compared to 53% by standard echo. On the other hand, cMRI has certain limits, especially in patients with metallic devices such as spinal rods in cases of operated scoliosis. Also, there is poor compliance of patients to undergo this test; in addition, the test has more limitations in young patients where sedation is needed for optimum results. However, considering cardiac MRI as a routine method in detecting CI would be justified clinically or cost-effective will depend on the answer to whether detecting minor abnormalities is a convincing factor to modify the protocol used in caring for these patients. Whether early diagnosis of CI has an implementation on the timing of commencing treatment and whether early treatment should be considered before the whole picture of DCM emerges is still to be answered.

We adhere to our institute guidelines regarding cardiac screening in BMD patients: cardiac evaluation with ECG and echo at diagnosis, follow-up at least every five years, or more frequent assessment in cases where progressive abnormalities emerge. This should be re-evaluated as some of the patients developed DCM in less than five years time.

Limitations of the study

The study was conducted in a retrospective manner. Some data was missing, which interfered with the interpretation of results. Although our clinical genetic unit is the only referral one in Jordan, we believe that not all Jordanian patients with BMD undergo regular follow-ups in the cardiac and clinical genetic clinics. Autonomic function assessment and ambulatory ECG monitoring were not included in our methods. Echo data is not included in this report and will be published separately.

## Conclusions

Cardiac involvement is part of dystrophinopathies in BMD patients. CI occurs usually without cardiac symptoms, and the severity is disproportionate to that of skeletal muscle. Abnormal ECG findings were found in 77%. Q wave and repolarization changes should be taken seriously as early signs of DCM, even if the echo is normal. We recommend cardiac screening as follows: at diagnosis, a two to three-year interval between each assessment seems justified if the initial tests were normal, and in case any abnormalities are detected by ECG or echo, yearly assessment should be considered. The routine usage of ECG and echo for screening is justified for the time being. Cardiac MRI should be considered in the surveillance of these patients.
